# Glucose metabolism in B cell malignancies: a focus on glycolysis branching pathways

**DOI:** 10.1002/1878-0261.13570

**Published:** 2024-01-03

**Authors:** Helga Simon‐Molas, Rosita Del Prete, Anna Kabanova

**Affiliations:** ^1^ Departments of Experimental Immunology and Hematology Amsterdam UMC location University of Amsterdam The Netherlands; ^2^ Cancer Immunology Cancer Center Amsterdam The Netherlands; ^3^ Fondazione Toscana Life Sciences Foundation Siena Italy

**Keywords:** B cells, glucose metabolism, T cells, tumor microenvironment

## Abstract

Glucose catabolism, one of the essential pathways sustaining cellular bioenergetics, has been widely studied in the context of tumors. Nevertheless, the function of various branches of glucose metabolism that stem from ‘classical’ glycolysis have only been partially explored. This review focuses on discussing general mechanisms and pathological implications of glycolysis and its branching pathways in the biology of B cell malignancies. We summarize here what is known regarding pentose phosphate, hexosamine, serine biosynthesis, and glycogen synthesis pathways in this group of tumors. Despite most findings have been based on malignant B cells themselves, we also discuss the role of glucose metabolism in the tumor microenvironment, with a focus on T cells. Understanding the contribution of glycolysis branching pathways and how they are hijacked in B cell malignancies will help to dissect the role they have in sustaining the dissemination and proliferation of tumor B cells and regulating immune responses within these tumors. Ultimately, this should lead to deciphering associated vulnerabilities and improve current therapeutic schedules.

## Graphical abstract

This review focuses on discussing general mechanisms and pathological implications of glycolysis and its branching pathways in the biology of B cell malignancies. We summarize here what is known regarding pentose phosphate, hexosamine, serine biosynthesis, and glycogen synthesis pathways in this group of tumors and in the tumor microenvironment.

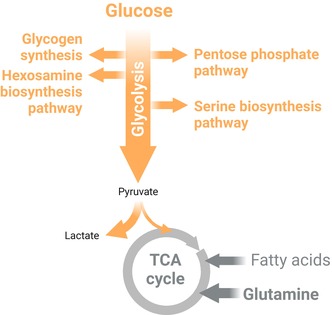

**Abbreviations**
3PG: 3‐phosphoglycerate
6PGL: 6‐phosphoglucolactone
B‐ALL: acute B cell lymphoma
BCR: B cell receptor
CLL: chronic lymphocytic leukemia
DLBLC: diffuse large B cell lymphoma
F6P: fructose 6‐phosphate
FL: follicular lymphoma
G1P: glucose‐1‐phosphate
G6P: glucose 6‐phosphate
G6PC: glucose‐6‐phosphatase
G6PD: glucose 6‐phosphate dehydrogenase
GFPT: glutamine:fructose 6‐phosphate amidotransferase
GSK3: glycogen synthase kinase 3
HBP: hexosamine biosynthesis pathway
HIF‐1α: hypoxia inducible factor‐1α
LN: lymph node
MM: multiple myeloma
MPC: mitochondrial pyruvate carrier
MPC: mitochondrial pyruvate carrier
mTOR: mammalian target of rapamycin
OGT: *O*‐GlcNAc transferase
OXPHOS: oxidative phosphorylation
PB: peripheral blood
PCK: phosphoenolpyruvate carboxykinase
PEP: phosphoenolpyruvate
PFK1: phosphofructokinase 1
PFK2: phosphofructokinase 2
PGM3: phosphoglucomutase 3
PHGDH: phosphoglycerate dehydrogenase
PPP: pentose phosphate pathway
PSAT1: phosphoserine aminotransferase 1
PSPH: phosphoserine phosphatase
SBP: serine biosynthesis pathway
TALDO: transaldolase
TCR: T cell receptor
TK: transketolase
TLR: toll‐like receptor 9
TME: tumor microenvironment
Treg: regulatory T cells
UDP‐GlcNAc: UDP *N*‐acetyl glucosamine

## 1 Introduction

Malignant cells are widely known for displaying peculiar features that distinguish them from normal cells. Most cancer cells exhibit accelerated cell cycle, invasive growth, increased cell migration, and elevated resistance to cellular stress. Sustaining these processes requires high energy expenditure and biosynthesis. Consequently, metabolic pathways in tumor cells are frequently rewired [1]. In the last two decades, glucose metabolism has become a crucial topic in the study of cancer biology, given that mechanistic bases of tumor plasticity strengthened by metabolic alterations have so far only been partially explored.

**Fig. 1:**
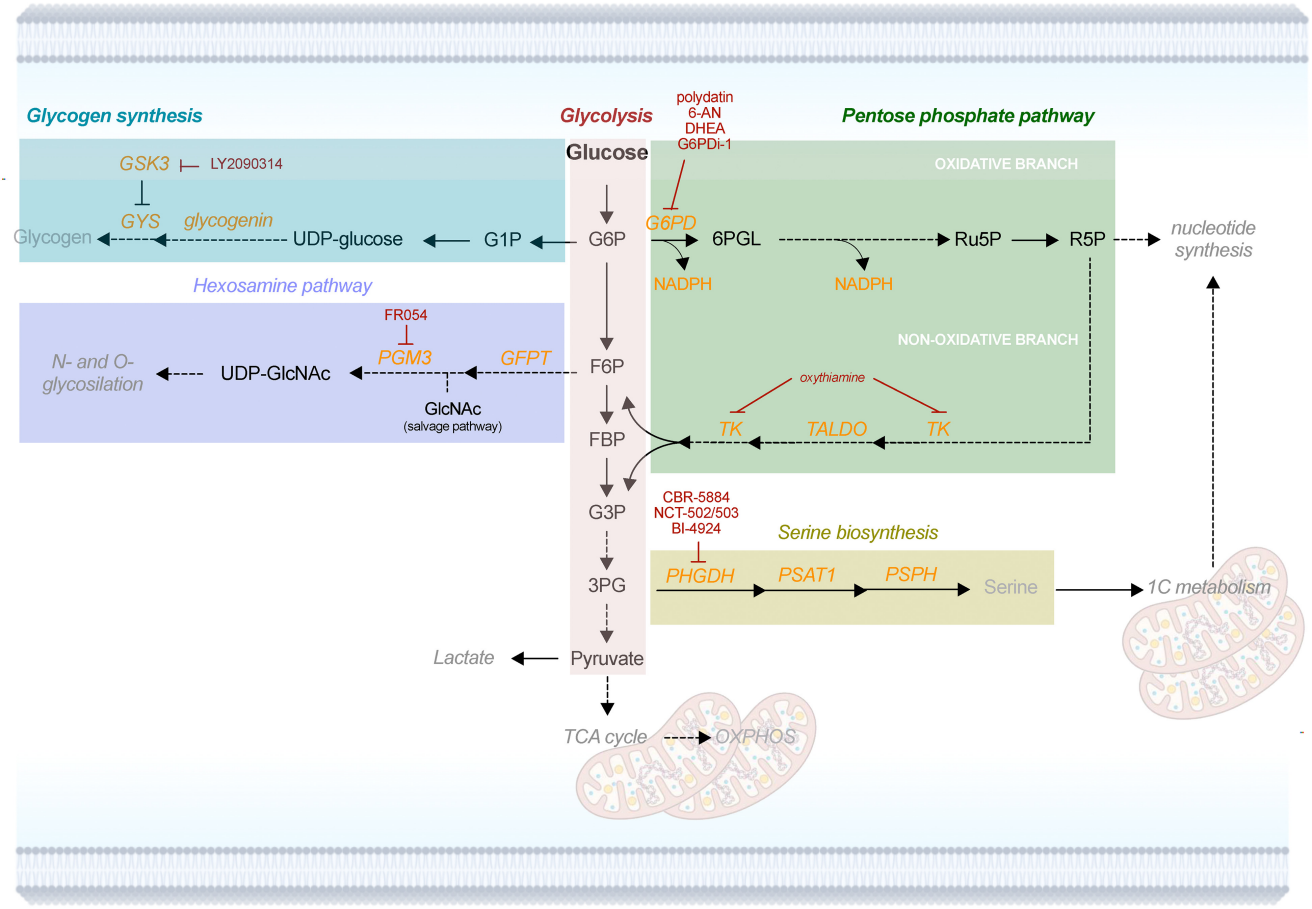
Schematic representation of ‘classical’ glycolysis and glycolysis branching metabolic pathways. Branching pathways are highlighted in colored boxes, including glycogen synthesis (blue), pentose phosphate pathway (green), hexosamine pathway (violet) and serine biosynthesis (yellow). Orange: main enzymes and cofactor(s); red: enzymatic inhibitors. Solid arrows represent direct metabolic reactions; dashed arrows represent a series of reactions. Created with Biorender.com. 3PG, 3‐phosphoglycerate; 6PGL, 6‐phosphoglucolactone; F6P, fructose 6‐phosphate; G1P, glucose‐1‐phosphate; G6P, glucose 6‐phosphate; G6PC, glucose‐6‐phosphatase; G6PD, glucose 6‐phosphate dehydrogenase; GFPT, glutamine:fructose 6‐phosphate amidotransferase; GSK3, glycogen synthase kinase 3; PHGDH, phosphoglycerate dehydrogenase; PSAT1, phosphoserine aminotransferase 1; PSPH, phosphoserine phosphatase; TALDO, transaldolase; TK, transketolase; UDP‐GlcNAc, UDP *N*‐acetyl glucosamine.

Glucose is a primary source for supporting cellular energy needs. Through glycolysis, glucose is converted into pyruvate, generating energy ‘currency’ (ATP and NADH) to be used in cellular processes with high energy demand. Pyruvate, the glycolysis end‐product, can be metabolized into acetyl‐CoA and incorporated into the TCA cycle, or converted into lactate. In most tissues, the first type of conversion is predominant in aerobic conditions, when oxygen is available, while lactate production occurs in anaerobic conditions. The first evidence of altered glucose metabolism in cancer was proposed at the end of the 1920s by Otto Warburg. His findings demonstrated a preferential skewing of glucose toward lactic acid fermentation at the expense of oxidative phosphorylation (OXPHOS) even when sufficient oxygen was available, a phenomenon known as aerobic glycolysis or the ‘Warburg effect’ [2]. For more than 10 years now, an altered metabolism has been considered a hallmark of cancer, and Warburg's observations have been expanded and extensively studied in several tumor types. Essentially, a varied milieu of fuels and metabolic pathways contribute to tumor progression, in each the tumor type [1, 3]. Besides glucose oxidation through glycolysis and the consequent production of acetyl‐CoA and lactate, increasing number of discoveries have recently elucidated the central role of *glycolysis branching pathways* in tumor growth and adaptation. These pathways divert glucose carbon flux at various levels of glycolysis into several anabolic pathways, including pentose phosphate pathway (PPP) and glycogen synthesis branching from glucose‐6‐phosphate, hexosamine pathway (HBP) from fructose 6‐phosphate, and serine biosynthesis (SBP) from 3‐phosphoglycerate (Fig. 1). Metabolites generated by these pathways feed into numerous metabolic processes and allow for particular degree of flexibility to tumor metabolic reprogramming (Box 1).

Box 1 **Description of glycolysis and glycolysis branching pathways**

Glucose can be metabolized not only through the ‘classical’ glycolysis, but also through glycolysis branching pathways, which include the pentose phosphate, hexosamine biosynthesis, serine biosynthesis, and glycogen synthesis pathways (Fig. 1).

- Glycolysis

Glycolysis is generally regarded as a central pathway of glucose catabolism within cells. Its initial substrate, glucose, is mostly internalized by cells from the extracellular media through different glucose transporters (GLUTs), the expression of which is tissue specific and increased in most cancers [4]. Through a series of nine reactions, glucose is converted into the end‐product pyruvate with the subsequent production of two ATP molecules. Pyruvate can enter the mitochondria and be converted into acetyl‐CoA, which fate is mostly the TCA cycle or lipid synthesis; or remain in the cytoplasm and be converted into lactate [5]. In conditions of low glucose availability, cells can obtain it through the breakdown of glycogen (glycogenolysis) and further metabolize it through glycolysis, or by synthesizing it through gluconeogesis, a process involving the same reactions of glycolysis in reverse direction with exception of three irreversible steps, which are specific for the biosynthetic pathway [6].

- Pentose phosphate pathway (PPP)

PPP stems from glycolysis at the glucose 6‐phosphate (G6P) node. It has a pivotal role in generating NADPH, which safeguards cellular redox status via reduced glutathione regeneration and is used for fatty acid and cholesterol biosynthesis [7], and ribose 5‐phosphate (R5P), the substrate for purine and pyrimidine biosynthesis. PPP consists of oxidative and non‐oxidative phases, fulfilling many requests of metabolic homeostasis. G6P enters PPP via a key rate‐limiting reaction by glucose 6‐phosphate dehydrogenase (G6PD) and is metabolized through a series of irreversible reactions, producing two NADPH and ribulose‐5‐phosphate (Fig. 1). The balance between oxidative (irreversible) and non‐oxidative (reversible) PPP phases is dynamically regulated through the activity of specific enzymes. If a cell is under oxidative stress and requires more NADPH, the metabolite flux can be re‐channeled toward the non‐oxidative PPP mediated via the activity of transketolase (TK) and transaldolase (TALDO). The resulting fructose 6‐phosphate (F6P) can be transformed back into G6P to generate additional NADPH molecules, in a sort of ‘NADPH salvage cycle’ (Fig. 1). On the other hand, if a cell has demand for increased nucleotide synthesis, the flux can be channeled in favor of R5P production.

- Hexosamine biosynthesis pathway (HBP)

HBP refers to a series of metabolic reactions in which fructose‐6‐phosphate (F6P) is converted into UDP *N*‐acetyl glucosamine (UDP‐GlcNAc), generating building blocks for N‐ and O‐linked glycosylation (Fig. 1). Hence, HBP plays a crucial role in post‐translational modifications of proteins and glycolipids. The first rate‐limiting reaction of HBP is mediated by glutamine:fructose‐6‐phosphate amidotransferase (GFPT).

- Serine biosynthesis pathway (SBP)

The SBP stems from the glycolysis at the level of 3‐phosphoglycerate (3PG), which is converted into serine through three enzymatic reactions catalyzed by phosphoglycerate dehydrogenase (PHGDH), phosphoserine aminotransferase 1 (PSAT1) and phosphoserine phosphatase (PSPH). In addition to being a precursor of several amino acids and sphingolipids, serine is also involved in nucleotide biosynthesis via one‐carbon metabolism [8].

- Glycogen synthesis

Glycogen synthesis refers to a series of enzymatic reactions allowing cells to store glucose in the form of glycogen, an activity that was originally thought to take place only in the liver. Glycogen is a complex glucose polymer with highly branched structure, in which glucose molecules are joined by α(1,4) glycosidic bonds and, around every 10 residues, by α(1,6) glycosidic bonds. Large molecular weight of the glycogen (up to 120 000 glucose residues, several mDa) allows glucose molecules to be stored without osmotic imbalance, which may happen in case of large quantities of free glucose monosaccharides. During glycogen synthesis, G6P‐derived glucose‐1‐phosphate (G1P) reacts with UTP to form UDP‐glucose (Fig. 1). Then, the enzyme glycogenin creates initial short glycogen chains, producing a sort of ‘primer’ for glycogen production, while the rate‐limiting enzyme glycogen synthase (GYS) extends the growing polysaccharide by adding glucosyl groups from the UDP‐glucose pool.

B cell malignancies account for most of the non‐Hodgkin lymphomas in the Western world and include different diseases such as chronic lymphocytic leukemia (CLL), diffuse large B cell lymphoma (DLBLC), follicular lymphoma (FL), multiple myeloma (MM) and acute B cell lymphoma (B‐ALL), among others. Study of B cell tumor metabolism is complex given the highly diversified ontogeny and heterogeneity in mutational and transcriptional profiles, even within the same tumor type (reviewed in detail elsewhere, [9, 10, 11, 12, 13, 14, 15]). Another critical factor that shapes B cell metabolic activity is their maturation status. Pro‐B cell and large pre‐B cell stages in the bone marrow environment are characterized by sustained cell proliferation, while the transition to mature states and exit into the circulation is characterized by cellular quiescence. Upon encountering antigens in the peripheral lymphoid organs and undergoing germinal center reaction, mature B cells switch to a proliferative phenotype, which is reverted again to non‐proliferative but highly secretory profile when they differentiate into plasma cells and home to bone marrow [16]. Given the origin of B cell tumors at different stages of these development, it is possible that they inherit associated lineage‐specific metabolic reprogramming and hence use glycolysis and branching pathways to a different extent. Understanding this complexity requires comprehensive characterization of glucose utilization by different types of B cell subsets and malignancies.

Furthermore, biology of the malignant B cells and their metabolism are strongly influenced by the tumor microenvironment (TME), which encompasses diverse niches, including bone marrow and secondary lymphoid organs (spleen, lymph nodes, tonsils). These sites are enriched in various cellular types, such as stromal cells and cells of the immune system, that intimately interact with malignant B cells, either sustaining their survival and proliferation or aiming at eliminating them. It is mostly in the lymph nodes (LN) where mature B cells receive pro‐survival and proliferative signals [17, 18], and we recently found that these microenvironmental stimuli are also inducing a rewiring of their metabolism [19]. Beyond the direct effect on the turnover and fitness of tumor cells themselves, glucose metabolism contributes to shaping the TME, as the function of other cell types is also supported by these pathways.

In this review, mechanistic aspects and functional output of glycolysis and branching pathways are discussed in the context of both healthy and malignant B cells, to provide readers with a comprehensive overview of what is known in the field about glucose metabolism in B cells. Given that this is a new field of research in B cells and that most translational studies involving glycolysis branching pathways have been performed on solid tumors, an overview of the main findings regarding the targeting of these pathways in solid malignancies is provided. In the last section, experimental work exploring glucose metabolism in the TME of B cell tumors is described, and possible mechanisms regulating metabolic equilibrium in the tumor niche are discussed.

## 2 Glucose metabolism in healthy B cells

### 2.1 Glycolysis in healthy B cells

Metabolic regulation of healthy B cell subsets has not been studied as extensively as in other immune cell subsets, and most of the work so far focused on mature follicular B cells. Nevertheless, peculiar features and metabolic preferences of B cells have started to emerge, mostly derived from studies in animal models.

B cells drastically change their metabolism during cell activation. Transition from quiescence to activation and proliferative burst, which leads to rapid cell divisions with approximately 9 h of cycling time [20], requires radical changes in gene transcription, promoting a metabolic adaptation necessary to match energetic demands of activated B cells. To achieve full metabolic capacity, it has been shown that B cells need to be stimulated not only through their B cell receptor (BCR), but also through secondary costimulatory signals and soluble cytokines that are pivotal for promoting full B cell activation *in vitro* and *in vivo*. For example, B cells respond to BCR triggering by rapidly increasing both glycolysis and OXPHOS [21]. However, without timely co‐stimulation through CD40 or TLR9, the glycolytic capacity and mitochondrial functionality of activated B cells are lost. Accordingly, in another study, it has been shown that glycolytic capacity of B cells grows proportionally with activation and might get exacerbated in B cells chronically overexposed to the stromal cell‐derived growth factor BAFF [22]. Altogether, these studies reveal a fine biochemical coupling between costimulatory signaling and growing metabolic needs of rapidly proliferating B cells.

**Fig. 2:**
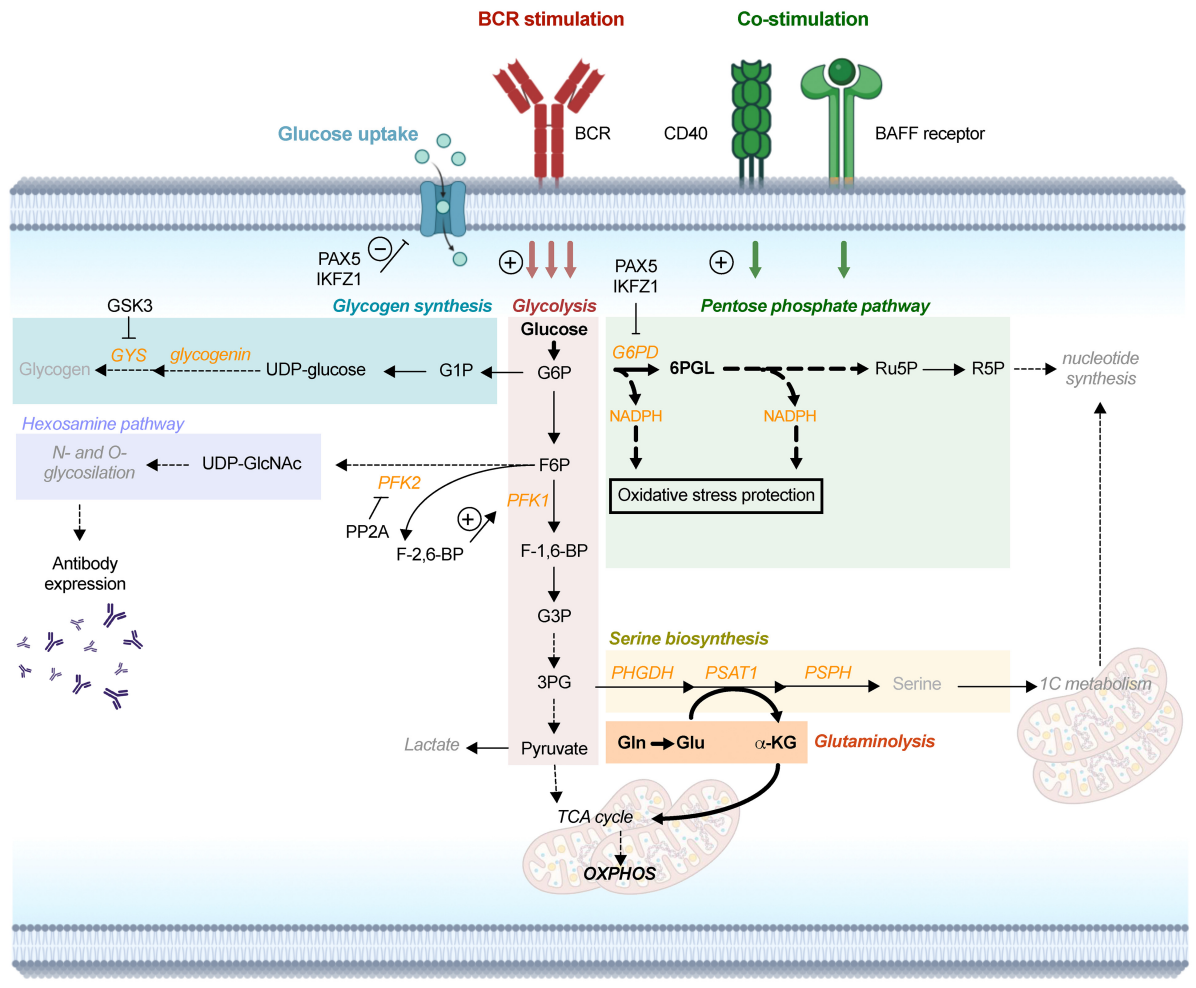
Glucose metabolism in healthy and malignant B cells. Metabolic fluxes preferentially engaged upon B cell stimulation (PPP and glutaminolysis‐TCA‐OXPHOS axis) are indicated in bold. Orange: main enzymes and cofactor(s). F‐1,6‐BP: fructose‐1,6‐biphosphate, F‐2,6‐BP: fructose‐2,6‐biphosphate. Solid arrows represent direct metabolic reactions; dashed arrows represent a series of reactions. Created with Biorender.com. 3PG, 3‐phosphoglycerate; 6PGL, 6‐phosphoglucolactone; BCR, B cell receptor; F6P, fructose 6‐phosphate; G1P, glucose‐1‐phosphate; G6P, glucose 6‐phosphate; G6PC, glucose‐6‐phosphatase; G6PD, glucose 6‐phosphate dehydrogenase; GFPT, glutamine:fructose 6‐phosphate amidotransferase; Gln, glutamine; Glu, glutamate; GSK3, glycogen synthase kinase 3; OXPHOS, oxidative phosphorylation; PFK1, phosphofructokinase 1; PFK2, phosphofructokinase 2; PHGDH, phosphoglycerate dehydrogenase; PSAT1, phosphoserine aminotransferase 1; PSPH, phosphoserine phosphatase; TALDO, transaldolase; TK, transketolase; UDP‐GlcNAc, UDP *N*‐acetyl glucosamine; α‐KG, alpha‐ketoglutarate.

Another interesting aspect is that B cell metabolism does not always strictly depend on the classic glycolysis‐TCA cycle axis. Initial steps of B cell activation through CD40 have been linked to a preferential flux of glucose toward the PPP and increased glutaminolysis to fuel OXPHOS, with little flux from glycolysis into the TCA cycle [23] (Fig. 2). It has been also shown that germinal center B cells conduct minimal glycolysis and tend to use fatty acid oxidation to fuel mitochondrial respiration [24]. The same metabolic preferences have been described for B1 cells and marginal zone B cells [25]. In conclusion, this suggests that an in‐depth study of metabolic rewiring induced by different stimuli in specific B cell subsets could help to unveil the complex metabolic picture governing B cell activation and functioning in health.

### 2.2 Glycolysis branching pathways in healthy B cells

The role of glycolysis branching pathways in healthy B cell compartment has been explored for several B cell subsets: pro‐B cells (mature B cell precursors), mature B cells, and plasma cells (terminally differentiated and long‐lived antibody producers). In pro‐B cells, antioxidant activity of PPP has been found essential to protect B cells from the oxidative damage [26]. Namely, the activity of the negative regulator of phosphofructokinase 2 (PFK2), PP2A, is essential to restrain glucose flux through glycolysis since PFK2 inactivation halts the positive feedback loop promoting activity of the glycolytic enzyme PFK1 (Fig. 2). This results in enforced diversion of glucose‐derived carbons into PPP [26]. Meanwhile, the contribution of the glycogen synthesis pathway to B cell regulation has been indirectly suggested by a study in which glycogen synthase kinase 3 (GSK3) has been described as a new metabolic checkpoint regulator in B cells [27]. Expression of GSK3, a negative regulator of GYS activity (Fig. 2) was found essential to constrain B cell over‐activation, hence suggesting that upregulation of glycogen synthase activity might be the underlying mechanism, although this has not been proved directly. Finally, in plasma cells, HBP plays a primary role in maintaining high rates of protein biosynthesis and glycosylation, protecting cells from unfolded protein response [28]. Overall, these studies collectively suggest that glycolysis branching pathways might play an important role in B cell regulation and function in health, although many aspects of this regulation have yet to be fully dissected.

## 3 Glucose metabolism in malignant B cells

### 3.1 Glycolysis in malignant B cells

Conversely to studies on healthy B cells, those on B cell tumors have been mostly performed in the context of primary human cells and have elucidated the contribution of glucose metabolism to a greater extent. In general, it has been proposed that glucose uptake plays an important role in regulating tumor B cell proliferation, which has been shown in studies on B‐ALL cells both *in vitro* [29] and *in vivo* [30]. Moreover, the latter study has proved that B‐lymphoid transcription factors, such as PAX5 and IKZF1, play a pivotal role in downregulating glucose uptake by B cells, hence having a metabolic gatekeeper function that poses necessary regulatory constraints upon B cell metabolism (Fig. 2).

The finding that glucose flux does not follow the classical glycolysis‐TCA cycle axis has been a cornerstone in our comprehension of malignant B cell metabolism. Almost 10 years ago it was discovered that CLL cells show an increased OXPHOS as compared to healthy B cells, albeit without significant differences in the production of lactate [31]. This observation is in line with the study of Waters et al. [23] on healthy B cells, discussed above. Further mechanistic insight into this reprogramming has been provided in the study of Le et al. on Burkitt lymphoma cell lines and our recent study on primary CLL cells showing that, upon stimulation, malignant B cells sustain their mitochondrial metabolism through glutaminolysis, instead of relying on the glycolytic end product pyruvate [19, 32] (Fig. 2).

### 3.2 Glycolysis branching pathways in malignant B cells

While glucose is still used to fuel glycolysis being converted into lactate, leukemic CLL cells divert a significant amount of its flux toward the PPP [19]. A transcriptomic signature of PPP activation has been observed as well in a mantle cell lymphoma 3D *in vitro* model [33]. Functional consequences of this rechanneling have been investigated for B‐ALL, where studies in murine models and patient samples have provided an in‐depth insight into the role of PPP in tumor cells. First, PPP has been shown to play a central role in protecting B‐ALL cells from oxidative stress. PAX5 and IKZF1 in such case were found to negatively regulate the expression of the rate‐limiting PPP enzyme G6PD (Fig. 2), hence confirming that B‐lymphoid transcription factors play a central role in regulating cellular metabolism. Second, pharmacological and genetic disruption of PPP2A activity has been shown to be detrimental for B‐ALL cell survival and leukemia development [26].

In parallel, SBP has been discovered as a crucial node regulating malignant B cell proliferation. A study by D'Avola et al. [34] showed that a functional SBP is sharply upregulated following B cell activation *in vitro*, and during the germinal center reaction *in vivo*. Human germinal center B cell‐derived lymphomas were found to overexpress high levels of the key SBP enzymes PHGDH and PSAT1 (Fig. 2). Accordingly, PHGDH inactivation by the small molecule PH‐755 inhibited proliferation of Burkitt lymphoma cell lines *in vitro* and blocked disease development in a Eu‐Myc lymphoma mouse model [34]. Beyond identifying a new potential therapeutic target for aggressive B cell lymphoma, this study interestingly suggested that certain branching pathways of glucose metabolism might be regulated by B cell activation and thus play role in relevant physiological processes.

Overall, findings describing how glycolysis branching pathways regulate physiologically relevant processes in healthy B cells and how they shape tumor aggressiveness in malignant B cells suggest that they might all represent potential therapeutic targets. As proof of principle, targeting glycolysis branching pathways has been explored at the preclinical level in various types of solid tumors (Box 2), and therefore, it is to be expected that further studies in the B cell field will also allow to apply this knowledge to translational approaches.

Box 2 **Targeting glycolysis branching pathways: knowledge from solid tumors**

- The pentose phosphate pathway, an exploited therapeutic target

As the rate‐limiting enzyme of PPP, G6PD plays a crucial role in cancer progression. Several studies suggested a correlation between high G6PD expression and prolonged survival of tumor cells, correlating with poor prognosis [35, 36, 37, 38, 39, 40, 41, 42, 43] and insurgence of drug resistance [24, 25, 26]. Metabolomics and stable isotope tracing revealed metabolic dependency of glioma cells on PPP‐generated nucleotides through G6PD overexpression [44]. In skin cancer, G6PD expression correlated with the presence of distant metastases during disease development and immune activity in the tissues affected, suggesting G6PD levels could be a biomarker predicting immunotherapy response [45]. Some cancer cells could adopt a PPP‐wired double protection mechanism to resist elevated oxidative stress mediated by reactive oxidative species (ROS). For instance, melanoma cells are dependent on G6PD to manage oxidative stress during metastasis [46, 47, 48, 49], albeit a compensatory metabolic reprogramming aiding ROS protection might arise as a consequence of G6PD deficiency [50].

On the other hand, G6PD silencing might contribute as well to tumor aggressiveness, albeit through a different mechanism. In colorectal carcinoma, loss of G6PD resulted in NADP accumulation and impaired nucleotide biosynthesis, arresting cell proliferation to favor cell survival [51]. Hence, G6PD activity might determine a dynamic balance between cell proliferation versus resilience under stress conditions, and the effect of PPP inhibition needs to be evaluated in a tumor‐specific context.

PPP is considered an important target for anti‐cancer treatment. The inhibition of G6PD by polydatin, a glucoside of resveratrol used for many years to treat different pathological conditions [52, 53, 54], induces endoplasmic reticulum stress and autophagy in breast cancer cells [55]. 6‐aminonicotinamide (6‐AN), an analogue of NADP that directly antagonizes G6PD activity, significantly decreased viability, colony formation capacity, and migration of breast cancer cells, leading to ROS upregulation and abnormal autophagy [56]. Ghergurovich et al. [57] demonstrated that the most widely cited G6PD antagonist, dehydroepiandrosterone (DHEA), did not robustly inhibit G6PD activity. Instead, they identified a small molecule (G6PDi‐1) that more effectively inhibited G6PD enzymatic activity, decreasing inflammatory cytokine production in T cells and suppressing respiratory burst in neutrophils. Despite TK is not a rate‐limiting enzyme of PPP, its role in cancer progression has been also considered important because of its ability to promote ‘NAPDH salvage’ (Fig. 1) [58, 59, 60, 61, 62, 63]. Targeting TK by genetic knockdown or pharmacologic inhibition by oxythiamine has been reported to increase oxidative stress of hepatocellular carcinoma cells, making them more vulnerable to different approved drugs, such as Sorafenib [64].

- The hexosamine pathway, a potential new target

Several findings have suggested the role of HBP in supporting cancer progression. Esophageal squamous cell carcinomas and adenocarcinomas exhibit increased expression of *O*‐GlcNAc transferase (OGT) and greater HBP activation compared to non‐neoplastic tissues [65]. Hepatocellular carcinomas display an enhanced global *O*‐GlcNAcylation levels due to the depletion of the gluconeogenic enzyme phosphoenolpyruvate carboxykinase (PCK) 1, which promotes enhanced cell proliferation and tumor progression [66]. Some studies also demonstrated a critical role of HBP in the metastasis of colon cancer and cholangiocarcinoma [67, 68], whereas elevated GlcNAc levels were found the strongest predictor and promoter of aggressiveness in systemic mastocytosis [69]. In MLL‐fusion leukemia, *O*‐GlcNAcylation of DOT1‐like histone H3K79 methyltransferase promotes oncogene expression and cell proliferation [70], hence suggesting HBP might control critical oncogenic mechanisms.

The first rate‐limiting enzyme of HBP, glutamine:fructose 6‐phosphate amidotransferase (GFPT), has been found overexpressed in several tumors [67, 71, 72, 73, 74, 75, 76, 77]. Others demonstrated that a decrease of GFPT expression might as well promote tumor development. For instance, in gastric cancer and melanoma, low GFPT levels correlated with bad prognosis [78, 79]. Hence, the consequences of modulation in GFPT levels and/or activity should be considered in a tumor‐specific context.

Several small drug compounds have been identified as possible HBP inhibitors, although with suboptimal specificity and associated toxicity *in vivo* [80, 81, 82]. Nevertheless, recently a novel compound FR054 has been found to inhibit phosphoglucomutase 3 (PGM3), one of the key enzymes of HBP (Fig. 1). FR054 induces substantial decrease in UDP‐GlcNAc levels, with a consequent impairment of cell proliferation, survival and migration in breast cancer cells [63]. FR054 was proved effective in reducing the growth of pancreatic cancer when administered in combination with the cytidine analogue gemcitabine [83].

- Serine biosynthesis, a target for specific tumors

Several findings suggest the role of serine metabolism in supporting tumor cell growth [84, 85, 86, 87]. In colorectal cancer, PHGDH inhibition cooperates with serine and glycine depletion to inhibit 1C metabolism, global protein synthesis and cancer growth [88]. In acute myeloid leukemia, genetic or pharmacological blockade of PHGDH suppresses tumor development and increases the sensitivity to chemotherapy [89]. In melanoma and breast cancer cells, blocking purine synthesis with methotrexate triggers a shunt of glucose‐derived carbons into the SBP and one‐carbon metabolism, promoting epithelial‐mesenchymal transition and metastatic colonization [90, 91].

SBP activity has a great impact on metabolic equilibrium, affecting the activity of multiple related pathways. On one hand, SBP activity regulates metabolite flux into the HBP. In a recent study, it has been shown that low PHGDH expression potentiates metastatic dissemination in breast cancer [92]. PHGDH interacts with the glycolytic enzyme phosphofructokinase 1 (PFK1), and the loss of this interaction activates HBP causing aberrantly enhances protein glycosylation thus promoting cell migration and tumor dissemination [76]. On the other hand, it has been reported that PHGDH inhibition induces alterations in nucleotide metabolism, even in the presence of abundant extracellular serine, affecting simultaneously both the PPP and TCA cycle [93]. Interestingly, simultaneous restoration of PPP and TCA cycle metabolism rescued cell proliferation during PHGDH inhibition, supporting notion that PHGDH may regulate the mass balance within central carbon metabolism and thus control overall flux of metabolized glucose [93, 94].

Owing to its unique function, PHGDH might be considered a promising target in cancer therapy, since it has been found overexpressed in several tumors [84, 85, 95, 96]. Several small drugs that inhibit PHGDH have been developed. A non‐competitive inhibitor of PHGDH, CBR‐5884, has been identified to block the *de novo* synthesis of serine in melanoma and breast cancer cells, being selectively toxic for cancer cells lines with high serine biosynthesis [97]. Other PHGDH inhibitors, NCT‐502 and NCT‐503 reduce the production of glucose‐derived serine in breast cancer and suppress the growth of PHGDH‐dependent cancer cells in culture and in orthotopic xenograft tumors [98]. NCT‐503 and BI‐4924, another PHGDH inhibitor [83], have been reported to decrease migration and metastatic dissemination of melanoma and breast cancer cells [90, 91].

- Glycogen synthesis, a pathway to further explore

Glycogen accumulation has been described to favor cancer cell survival [84, 85, 86, 87, 88, 89] and metastasis [99, 100, 101, 102, 103]. Moreover, it has been proposed that glycogen accumulation might be a key initiating oncogenic event, playing a crucial role in malignant transformation. For instance, in liver, cancer glucose‐6‐phosphatase (G6PC), an enzyme catalyzing the last step of glycogenolysis, is frequently downregulated in pre‐malignant cells. This results in the accumulation of glycogen and activation of Yap kinase that promotes cell survival and transformation. Conversely, elimination of glycogen accumulation has been shown to abrogate cancer incidence, highlighting that cancer‐initiating cells might be distinguished by the ability to store glycogen [104].

## 4 Glucose metabolism in the microenvironment of B cell tumors: Glycolysis branching pathways as a new field of research

### 4.1 The TME in B cell malignancies

Biology of the TME is as important for the development and progression of tumors as that of malignant cells themselves. Interactions within TME cells (malignant, endothelial, stromal, and immune cells), the extracellular matrix and soluble factors including the composition of metabolite milieu confer an environment that sustains tumor growth [105]. Despite most studied cells in TME are, by far, tumor cells, studies focusing on other cellular compartments have increased over the last years. When it comes to metabolism, the picture is similar: the interest in metabolic pathways emerged first in the cancer field and it was not until the beginning of this century that metabolism started to be also explored in the TME, mostly dissecting its impact on the fate of immune cells and anti‐tumor immune response. Currently, immunometabolism is a research field on its own, and the number of publications in the topic has been increasing every year.

**Fig. 3:**
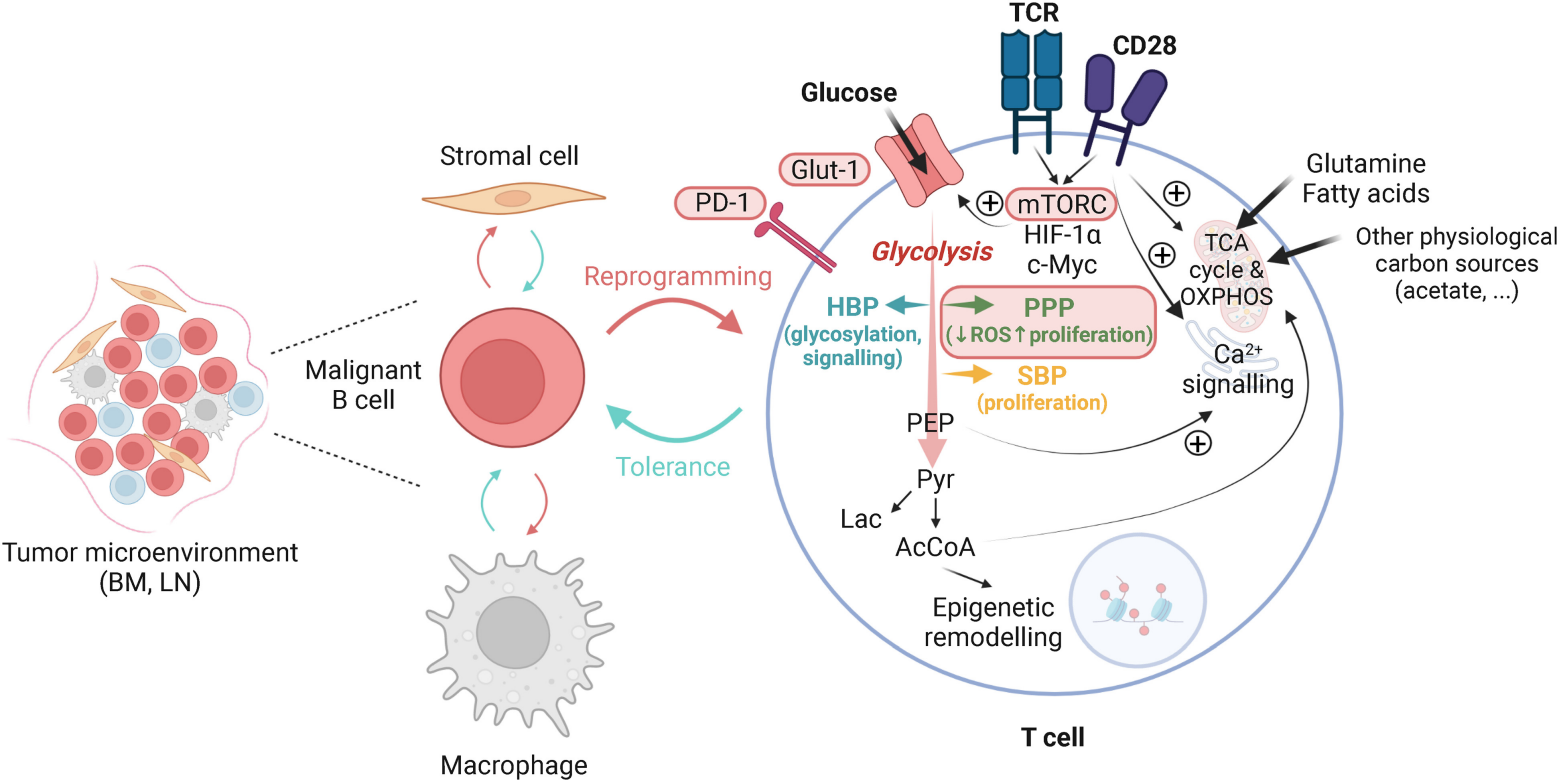
T cell glucose metabolism in B cell malignancies. Schematic representation of the cellular components of the TME in B cell tumors with a focus on T cell glucose metabolism. Depicted metabolic pathways have been described in healthy T cells or T cells in tumor context. Glycolysis is shown in red; glycolysis branching pathways are color‐coded according to previous figures. Red squares: metabolic components described to be altered in T cells in B cell malignancies. Pyr: pyruvate, Lac: lactate, AcCoA: acetyl‐CoA, Antiox: antioxidant. Solid arrows represent direct metabolic reactions; dashed arrows represent a series of reactions. Created with Biorender.com. AcCoA, Acetyl‐CoA; BM, bone marrow; GLUT1, glucose transporter; HBP, hexosamine biosynthesis pathway; HIF‐1α, hypoxia inducible factor‐1α; Lac, lactate; LN, lymph node; mTOR, mammalian target of rapamycin; OXPHOS, oxidative phosphorylation; PB, peripheral blood; PD‐1, Programmed cell death protein 1; PEP, phosphoenolpyruvate; PPP, pentose phosphate pathway; Pyr, pyruvate; SBP, serine biosynthesis pathway; TCR, T cell receptor.

In B cell malignancies, the role of the TME is currently a matter of investigation. Although it is clear that bone marrow and secondary lymphoid tissues are altered [17, 18, 106, 107, 108, 109], the specific function and contribution of each cell type to tumor progression is not completely understood yet. A common trait in B cell malignancies is the immunosuppressive component of the TME that consists of elevated numbers of exhausted T cells, regulatory T cells (Treg) and anti‐inflammatory macrophages with M2 phenotype (Fig. 3). Inhibitory cytokine production by tumor cells, M2‐like macrophages, myeloid‐derived suppressor cells, Tregs and stromal cells altogether impose immunosuppression by decreasing antigen presentation by tumor cells or by directly dampening the function of immune cells. Other mechanisms such as ROS and nitric oxide production have also been described to contribute to tumor immunosuppression, as extensively reviewed before [110]. When it comes to the stromal component of TME, a direct transfer of signaling molecules and even mRNA between cells can shape their phenotype, as it was described for CLL‐ and B‐ALL‐derived extracellular vesicles that reprogrammed TME stromal cells [111, 112]. It has also been observed that exosomes from stromal cells can trigger the glycolytic switch in CLL cells [113], suggesting a possibility for reciprocal conditioning. More recently, a study performing transcriptomic analyses at the single cell level from CLL has evidenced that gene expression of leukemic cells is shaped by the TME, and thus it is different in the PB compared to LN, where proliferation and metabolism signatures were enriched [114]. The authors showed that expansion of a specific clone in CLL occurs in the LN and is associated with increased presence of M2 macrophages and activated CD4^+^ memory T cells, with overall suppressed T cell inflammatory response, what they refer to as permissive TME [114].

As evidenced in several solid tumors, alterations in the metabolism of immune cells imposed by malignant B cells underlie immunosuppression [115]. In this section, we will summarize how glucose metabolism regulates the metabolic status of TME in B cell malignancies, with a focus of T cells, and draw parallels with the most recent discoveries from the field of healthy T cell biology and solid tumors, which might be extrapolated to B cell cancers.

### 4.2 Glucose metabolism in healthy T cells

The field of T cell metabolism has mainly explored the ‘classical’ glucose metabolism routes, including aerobic glycolysis through lactate production and utilization of acetyl‐CoA for the TCA cycle. It has already been known for decades now that, upon T cell receptor (TCR) ligation, quick downstream signaling cascades converging onto Akt phosphorylation drive the translocation of GLUT‐1 to the cellular membrane, causing a rapid increase in glucose uptake upon T cell stimulation. This was first shown by Rathmell et al. [116] by analyzing the mRNA expression of GLUT‐1 in mouse T cells, which was increased from 6 h after anti‐CD3/28 stimulation. The similarities between insulin signaling in fat and muscle cells and the TCR‐driven upregulation of glucose metabolism reported in this primary study evidenced that many of the regulatory mechanisms described until the moment in other cell types would be applicable also to T cells. Two years later, it was described how T cell co‐stimulation through CD28 led to increased glycolytic flux upon TCR engagement, which involved phosphatidylinositol 3′‐kinase (PI3K) and Akt [117]. More recent work confirmed the essential role of GLUT‐1 for the effector function of murine CD4^+^ T cells, specifically for Th1 and Th17 cells, and not for resting CD4^+^ T cells [118]. The glycolytic switch, which is mostly driven by coordinated activity of the mammalian target of rapamycin complex (mTORC), the hypoxia inducible factor‐1α (HIF‐1α) and c‐Myc transcription factors, is essential for the transition of naive T cells to effector cells, in order to fulfill their energy requirements for cytokine production and rapid proliferation during expansion [119] (Fig. 3).

The importance of the so‐called glycolytic switch upon TCR engagement does not account for ATP production only. By using metabolomics and stable isotope tracing with ^13^C‐glucose, a recent study has shown that a substantial fraction of glucose internalized by T cells is used for glycolysis branching pathways. Namely, these include the PPP, HBP, SBP, and one‐carbon metabolism, which are essential to neutralize ROS, synthesize nucleotides and maintain the glycosylation of lipids and proteins [120] (Fig. 3). This study showed that glucose diversion into SBP occurs mostly *in vivo*, while *in vitro* most of the serine is obtained from the extracellular media [120, 121]. Importantly, T cells stimulated in media with glucose concentration similar to plasma and supplemented with additional physiological carbon sources such as acetate, citrate, lactate, and pyruvate mostly used glucose to fuel upper glycolysis and the PPP. However, contribution of glucose to lower glycolysis and TCA cycle was clearly decreased in plasma‐like medium compared to culture conditions with the high glucose concentration that is usually used for *in vitro* cultures [122]. This finding points out that, at physiological conditions or *in vivo*, glycolysis branching pathways might be more relevant than the contribution of glucose to the TCA cycle or lactate production in T cells, contrary to what is usually observed *in vitro* (Fig. 3).

Following the glycolytic switch, a second metabolic adaptation takes places in T cells approximately 24 h after TCR stimulation and involves an increase in TCA cycle activity and OXPHOS to increase energy production and provide substrates for lipid and amino acid synthesis, essential elements for proliferation [119] (Fig. 3). Although it is widely assumed that mitochondrial metabolism is more relevant for memory T cells, whereas glycolysis is more important for T effector cells [123, 124, 125, 126], several studies have shown that both cell types require glycolysis and OXPHOS for their specific functions, energy production and proliferation. However, the fuels that mostly contribute to OXPHOS in each T cell type differ. In memory T cells, the predominant source of TCA cycle intermediates are fatty acids and amino acids, whereas glucose is assumed to be the most important contributor in effector T cells [127, 128]. In fact, it was shown that sustained glycolysis can co‐exist with the development of CD8 T cell memory phenotype during viral infections [129]. Other work showed that distinct CD4 T cell subsets use different metabolic pathways to support their function, with T helper 1 (Th1), Th2, and Th17 cells mostly engaging glycolysis, and regulatory T cells relying on fatty acid oxidation [130]. Later work has shown that proliferating Tregs also increase glycolysis and lipogenesis upon stimulation and are highly plastic according to nutrient availability conditions [131]. This capacity for adaptation can explain their persistence in hostile tumor environments. Flexibility of effector T cells was also demonstrated in mouse CD8^+^ T cells, which upregulated glutaminolysis and OXPHOS upon glucose withdrawal in an AMPK‐dependent manner [132]. Despite compensation with glutamine, cytokine production and proliferation were decreased in the absence of glucose, highlighting that T cells rely on extracellular glucose supply to accomplish their effector functions.

Beyond the strictly biochemical effect that glycolytic intermediates have on metabolic pathways, some of them have been shown to be crucial for proper signaling in T cells. This is the case of precursors of epigenetic modifications, and metabolites that can trigger signaling cascades. Several studies have reported that intracellular concentrations of acetyl‐CoA, which is required for histone acetylation, are highly dependent on the breakdown of glucose into pyruvate during glycolysis in T cells [133, 134]. This might provide an explanation to the unique histone acetylation landscape that differentiates effector CD8 T cells from exhausted T cells, which show impaired glycolytic rate [135]. Wenes et al., [128] demonstrated that inhibition of mitochondrial pyruvate carrier (MPC) promotes the utilization of fatty acids and amino acids in the TCA cycle and improves mitochondrial function, while allowing glucose‐derived acetyl‐CoA to be used for histone acetylation, which increases the expression of genes related to memory formation. Very recently, a connection between the expression of T cell activation markers upon TCR engagement and the glycolytic switch has been dissected, with glucose‐derived acetyl‐CoA production in the nucleus being responsible for histone acetylation of the promoter region of CD25 and other surface molecules and effector cytokines in the first hours after TCR engagement [136] (Fig. 3).

Another example of the role of glycolytic intermediates in shaping T cell function is the evidence that phosphoenolpyruvate (PEP) is necessary for maximal Ca^2+^‐NFAT signaling in T cells. In highly glycolytic tumors, glucose availability in the TME can be insufficient and therefore T cells might show suboptimal Ca^2+^ signaling due to insufficient PEP [137] (Fig. 3).

### 4.3 T cell glucose metabolism in B cell malignancies

T cells from patients with B‐ALL or CLL show defective metabolism upon *in vitro* stimulation, with decreased Akt/mTORC1 signaling, reduced expression of GLUT‐1 and hexokinase 2, and decreased glucose metabolism, accompanied by dampened proliferation and increased expression of the exhaustion marker PD‐1 [138]. The same metabolic profile was observed in leukemic mice, where the authors also identified an increased expression of inhibitory ligands, such as PD‐L1, in the spleen. Leukemic mice with T cell‐specific transgenic expression of constitutively active Akt showed restored T cell metabolism and function, which contributed to a decrease in tumor burden, whereas anti‐PD1 therapy did not promote this effect. This study provided the first mechanistic explanation of the phenomenon of T cell dysfunction in B cell malignancies from a metabolic perspective [138] (Fig. 3). The mechanisms by which tumor cells alter and condition the metabolic status of the TME are not necessarily exclusive to tumors and can be recapitulated in healthy cells. In line with this, a recent study has shown that healthy B cells stimulated with CpG, anti‐IgG/IgM and IL‐2 increase their OXPHOS activity and suppress T cell effector function by inducing hypoxia and decreasing mTORC signaling through glucose deprivation and lactic acid production [139]. The metabolic status of B cells described in this article is very similar to that of activated CLL cells, which has been discussed in the previous section [19], and the dysfunction induced on T cells shares some similarities with that observed in patients with B cell malignancies.

Our group showed that T cell dysfunction in CLL patients is not only glycolysis‐ but also mitochondria‐driven, as T cells from these patients fail to increase their mitochondrial mass upon stimulation, as compared to T cells from age‐matched healthy individuals [140] (Fig. 3). This can not only dampen the activity of the TCA cycle and OXPHOS, but also of other key pathways that take place in the mitochondria such as one‐carbon metabolism, which is intrinsically linked to SBP. Besides, CLL T cells showed increased ROS levels and decreased expression of NRF2, which is a key antioxidant transcription factor that regulates, among others, the expression of key enzymes of the PPP and SBP [141]. In two follow‐up studies, we have shown that impaired OXPHOS [142], pseudohypoxia and adenosine signaling [143], contribute to at least part of the observed T cell dysfunction in CLL. Importantly, T cell dysfunction was reverted by depletion of CLL cells *in vitro* and in patients following treatment with venetoclax and obinutuzumab. Treated patients showed restored T cell activation and upregulation of GLUT‐1 expression, paralleled by a decrease in Treg percentage in peripheral blood [142].

Whether metabolic deficiencies also impact epigenetic modifications or calcium signaling and contribute to sustaining the immunosuppressive TME in patients with B cell malignancies is currently under investigation by our groups and others.

## 5 Conclusions, challenges, and perspectives

In this review, we discussed the role of glucose metabolism in healthy and malignant B cells, with a specific focus on glycolysis branching pathways. Clear evidence of their relevance in the pathogenesis of B cell tumors has emerged, however the number of studies conducted in hematological malignancies is far less than those performed in solid tumors.

One of the major challenges when studying B cell metabolism is that the function of metabolic pathways might be not homogeneous throughout the B cell compartment, with location (niches) and time (phases) being crucial components in determining their functional implications across the B cell life cycle. What regulatory networks account for rapid metabolic re‐programming underlying heterogeneous behavior of B cell subsets is still not fully understood. Both healthy and malignant B cells undergo metabolic reprogramming upon primary and secondary stimulation through BCR, Toll‐like receptors (TLRs), and CD40. One of the most important lessons learned in the last few years is that, contrary to what is observed in other proliferating cells, glucose flux in activated B cells is preferentially re‐routed into PPP or converted into lactate, whereas TCA cycle and OXPHOS are mostly fueled by glutamine. Specific enzymes favoring the PPP, such as PP2A, have been identified as metabolic dependencies in malignant B cells, and therefore glycolysis branching pathways are currently being investigated by our groups and others as new potential therapeutic targets. Research focusing on serine and hexosamine biosynthesis pathways in B cell tumors are less advanced. Nevertheless, several hints from the solid tumor field indicate that glucose utilization through these routes is also highly relevant for cancer progression.

Both B cells and T cells in the TME rely on glucose metabolism, including glycolysis branching pathways. Despite most evidence so far is based on expression data of PPP, SBP and HBP‐related enzymes, projects by us and others are aiming to unveil how glucose‐derived carbons are used by T cells, and which functions they underlie. Expanding these studies to other TME cellular components, such as tissue‐resident macrophages and stromal cells, would also be highly informative.

The fact that healthy and malignant B cells might have similar reprogramming of glucose metabolism poses a high threshold for the selection of tumor‐specific therapies targeting these pathways. If T cells are included in the picture, this threshold becomes even higher. However, the problem of therapy‐induced B cell aplasia would be the same in such case that anti‐CD19/20 immunotherapies and CAR‐T cell therapies face nowadays. If molecules inhibiting glycolysis branching pathways were to be administered in patients, it may be reasonable to accept depletion of the whole B cell compartment at the first phase of treatment, while aiming to restore the immune system of these patients at the second phase, once malignant cells have been eliminated. Besides the challenges discussed, several questions emerge such as (a) How related the flux through glycolysis branching pathways to disease drivers is (e.g. common mutations and chromosomal aberrations) and to the emergence of resistance; and (b) How valid the comparison of studies performed in healthy B cells, (mostly in mice) and human malignant B cells is, given the differences between these two species. Overall, although we are still far from having therapeutic solutions targeting glycolysis branching pathways in B cell malignancies, the findings of these last 5 years show us that these pathways are very relevant for B cell biology. We postulate that there is a rationale to invest efforts and funds to further develop the studies discussed in this review.

## Author contributions

HS‐M, RDP and AK conceived and wrote the manuscript.

## Conflict of interest

The authors declare no conflict of interest.
